# Advanced redox flow fuel cell using ferric chloride as main catalyst for complete conversion from carbohydrates to electricity

**DOI:** 10.1038/s41598-017-05535-2

**Published:** 2017-07-11

**Authors:** Fan Xu, Huan Li, Yueling Liu, Qi Jing

**Affiliations:** Key Laboratory of Microorganism Application and Risk Control of Shenzhen, Graduate School at Shenzhen, Tsinghua University, Shenzhen, 518055 China

## Abstract

Liquid catalyzed fuel cell (LCFC) is a kind of redox flow fuel cell directly converting carbohydrates to electricity. To improve its efficiency, ferric chloride (FeCl_3_) was introduced as main catalyst. As mono catalyst, phosphomolybdic acid (PMo_12_) was much better than phosphotungstic acid (PW_12_) and FeCl_3_ was intermediate between them. Compared with PMo_12_ at the optimal dose of 0.30 mol/L, the combination of FeCl_3_ (1.00 mol/L) and PW_12_ (0.06 mol/L) achieved similar power output from glucose (2.59 mW/cm^2^) or starch (1.57 mW/cm^2^), and even improved the maximum power density by 57% from 0.46 to 0.72 mW/cm^2^ when using cellulose as the fuel. Long-term continuous operation of the LCFC indicated that carbohydrates can be hydrolyzed to glucose and then oxidized stepwise to carbon dioxide. At the latter stage, there was a linear relationship between the electron transfer number from glucose to catalyst and the subsequent cell performance. Based on these findings, the contribution of FeCl_3_ to LCFC should be derived from the accelerated hydrolysis and oxidation of carbohydrates and the enhanced electron transfer from glucose to anode. The addition of FeCl_3_ reduced the usage of polyoxometalates by 80%, and the replacement implied that LCFC can be operated less toxically and more economically.

## Introduction

Bioenergy has been recognized as a key contributor to a sustainable society because of annual great yield of biomass and biomass waste. For example, the National Energy Administration of China reported that biomass waste could provide energy equivalent to 4.6 hundred million tons of standard coal annually in China, but only 5% of the biomass waste had been actually utilized^1^. Hence, bioenergy has great potential as a renewable energy source. Biomass can be converted to heat, electricity or fuels through different approaches including direct incineration, liquefaction, pyrolysis, gasification and anaerobic digestion. Compared with these conventional ways, fuel cells can convert biomass to electricity with higher energy efficiencies, but most of them can only utilize simple micro molecular fuels like hydrogen and methanol. For example, phosphoric acid fuel cells (PAFCs), molten carbonate fuel cells (MCFCs) and solid oxide fuel cells (SOFCs) have been used for commercial applications, but they only deal with hydrogen, methane or carbon monoxide at high operational temperatures and biomass has to be decomposed or reformed to these simple fuels before fed into SOFCs^2–5^.

For complex biomass utilization and organic waste treatment, those fuel cells directly consuming biomass under mild conditions are very valuable and have been developing rapidly in recent years. Alkaline fuel cells (AFCs) or alkaline anion exchange membrane fuel cells (AAEMFCs) can be fueled by glucose and even cellulose, but these chemicals cannot be thoroughly decomposed^6–8^. Microbial fuel cells (MFCs) are capable to fully degrade large molecular organic compounds under mild conditions^9, 10^, but some types of shock loading or toxics into MFCs would probably depress microbial activity during biomass waste treatment. Some kinds of proton exchange membrane fuel cells (PEMFCs) can also directly utilize complex biomass for electricity generation. For example, an intermediate-temperature (75‒250 °C) fuel cell using phosphoric acid as catalyst can convert wood sawdust and pulp to carbon dioxide with output power density of 10 mW/cm^2^ ^11^. Alternatively, a novel liquid catalyzed fuel cell (LCFC) was also proposed with the assistance of polyoxymetalates (POMs)^12^, and it performed well in generating electricity with nature polymeric biomasses^13, 14^. In fact, direct liquid redox fuel cells have been investigated in 2008 as an improvement to direct methanol fuel cells for numbers of advantages^15, 16^. Most of all, this type of fuel cells allowed the use of three-dimensional electrodes and consequently increased the reaction area. LCFCs share a similar structure with liquid redox fuel cells, and POMs work as both homogeneous catalysts and charge carriers in catholyte and anolyte.

The conversion from biomass to electricity in a LCFC includes three main steps^13^. Biomass was first oxidized by a type of POM (noted as POM-I) in anolyte, and then the reduced POM-I was re-oxidized during the cell reaction between anolyte and catholyte. In catholyte, oxygen was directly supplied or another kind of POM (noted as POM-II, relatively higher electrode potential than POM-I) was used as the electron carrier between oxygen and cathode. POMs are fine catalysts for the oxidation of biomass and the hydrolysis of macromolecule organics^17^. With its help, starch and cellulose can be hydrolyzed to glucose and then oxidized to carbon dioxide in LCFCs^12, 13^. Moreover, unlike other fuel cells, LCFCs use POMs for homogeneous catalysis instead of loading noble metal catalysts and they are insensitive to most contaminants. Due to its efficiency and adaptability, high output power density of 51 mW/cm^2^ was achieved even using bush allamanda as the fuel at the operating temperature of 80 °C and the condition of sunlight^12^. The high power output and the complete degradation of complex organic compounds implied its promising application in the field of organic waste treatment.

Although limited works on LCFCs have been carried out, optimization and improvement are still necessary for its further practical application to biomass waste treatment. The current LCFCs use POMs as catalysts, which are commonly complex, toxic and relatively expensive. Hence, in this study, Lewis acids were introduced as co-catalysts, aiming to replace most POMs and also enhance the degradation of complex carbohydrates. Glucose was first chosen as the model substance to conclude the optimal operational condition of LCFC and compare the capabilities of different POMs and Lewis acids. After that, the degradation abilities of glucose, starch and cellulose in LCFCs were analyzed. Based on the degradation process of carbohydrates and the electron transfer process in LCFC, the function of the added Lewis acid in LCFC was deduced. Finally, we developed a new type of LCFC using ferric chloride to replace most of complex POMs as co-catalyst, which is simpler, cheaper and more effective in degrading complex carbohydrates.

## Materials and Methods

### Construction and operation of LCFC

**Figure 1:**
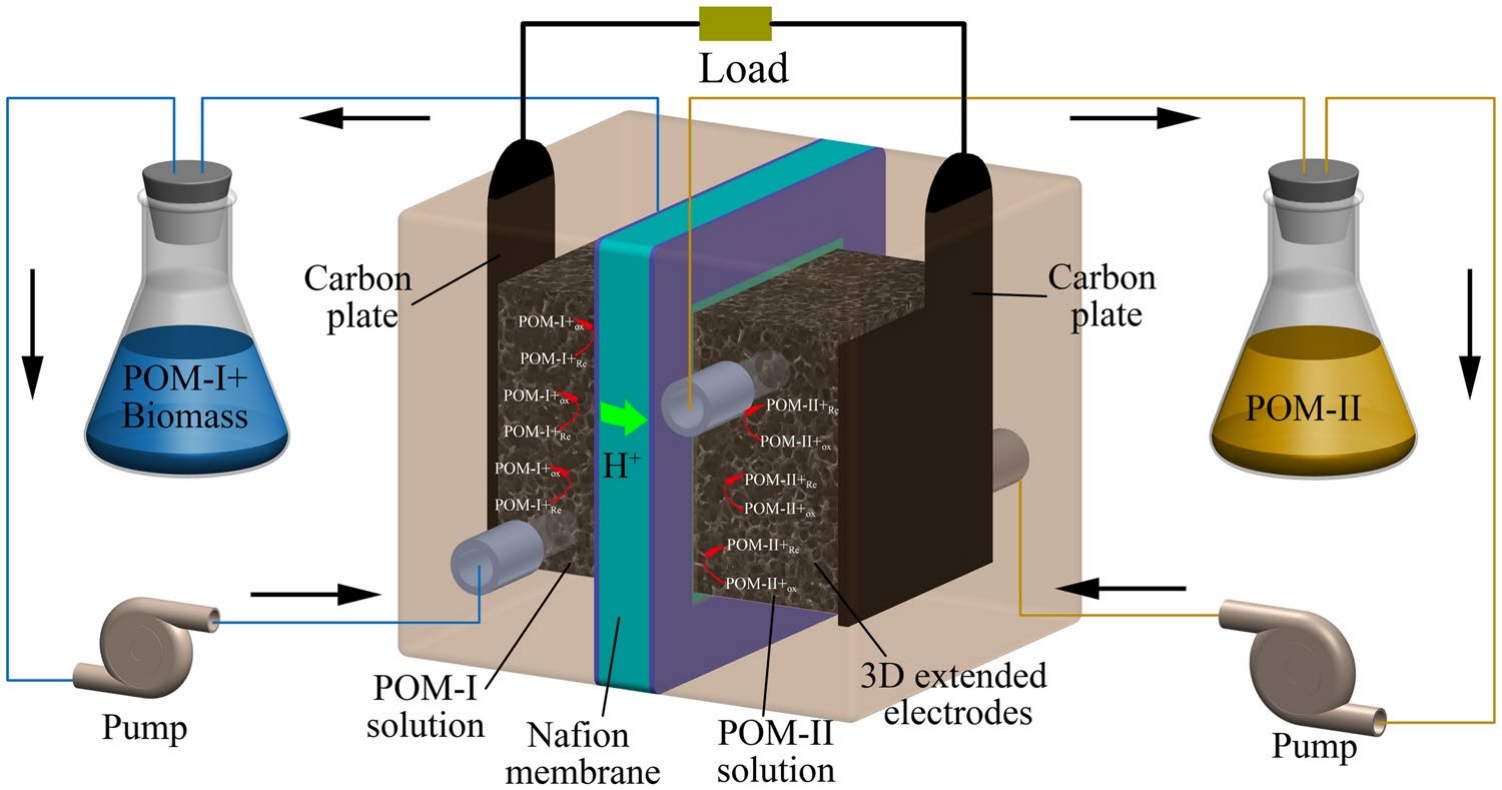
Structure of the LCFC.

The structure of the LCFC studied herein combined some features of PEMFCs and redox flow batteries (Fig. 1). Both the anolyte and the catholyte were stored in two electrolyte tanks and pumped into the fuel cell using peristaltic pumps. The cell was separated by a proton exchange membrane (Nafion 115, Dupont, USA) and it was sandwiched by two graphite felts as 3D extended electrodes. The sandwich was clamped by two graphite plates (Hongjun, Shanghai, China). All the contents were inset into an acrylonitrile butadiene styrene (ABS) plastic shell. There was a square-shaped channel on the shell with 7 mm deep, 20 mm long and 20 mm wide, and the graphite plat was fixed on the bottom of the channel and a part of it stretched out of the shell for exporting the current. The graphite felt with a thickness of 10 mm was loaded in the channel, fitting closely with the graphite plate. A gasket was put between the shell and the proton exchange membrane to prevent liquid leakage. The thickness of the gasket was 2 mm so the place for the graphite felts was totally 9 mm in depth considering the depth of the channel itself. Thus, the graphite felts were compressed by approximately 10%, so that they had a relative low resistance.

In order to clean the Nafion 115 membrane, it was pretreated in sequence in boiling solution of 3% H_2_O_2_ for 1 h, in boiling deionized water for 1 h, in boiling solution of 1 mol/L H_2_SO_4_ for 1 h and finally in boiling deionized water for 1 h. The 3D extended electrodes were activated to improve their hydrophilia. At first, the graphite felt was cut to suitable size and washed three times using deionized water in an ultrasonic cleaner (40 KHZ, KQ-400D, Nanbei Ltm., Zhenzhou, China) for 30 min. The cleaned graphite felt was dried in an air dry oven (DHG-9053A, Yiheng Ltm., Shanghai, China) at the temperature of 180 °C for 8 h, and then baked at 420 °C with dry air inlet in a muffle furnace (SX-G01123, Zhonghuan, Tianjin, China) for 10 h. Finally the carbon felt was cooled to ambient temperature (23‒25 °C).

The anolyte used phosphomolybdic acid (H_3_PMo_12_O_40_, PMo_12_) or phosphotungstic acid (H_3_PW_12_O_40_, PW_12_) as POM-I. The former was purchased from Aladdin, USA and the latter was purchased from Sigma, USA. Glucose (Maklin, Shanghai, China) was used as the model compound to investigate the performance of the LCFC. Calculated quantities of POM-I and glucose were dissolved in deionized water (0.30 mol/L POM-I and 2.00 mol/L glucose), and the pH of the solution was adjusted to about 0.1 using concentrated phosphoric acid (85 wt. %, Aladdin, USA) and the concentration of phosphoric acid was 3.00 mol/L. The prepared mixture was placed in a water bath with the temperature of 95 °C for 2 h, and then taken out for natural cooling to ambient temperature (25 °C) in a dark box. The cell was developed aiming to deal with real organic waste or wastewater, and thus we tried to investigate its performance at ambient temperature without heating. When using different Lewis acids replace some POM-I, phosphoric acid of 1.50 mol/L was adopted to keep the acidity for FeCl_3_ and CuSO_4_, while sulfuric acid of 1.50 mol/L was adopted for VOSO_4_ to avoid the precipitation of V^4+^ and V^3+^.

The catholyte used non-Keggin-type molybdovanadophosphoric acid (H_12_P_3_Mo_18_V_7_O_85_, P_3_Mo_18_V_7_) as POM-II, which was synthesized referring to the method reported before^18^. Unlike conventional Keggin-type POM, this kind of non-Keggin-type POM contains more vanadium atoms in a molecule. For the synthesis, 1200 ml deionized water was kept at 0 °C by ice water mixture, and a magnetic stirrer was applied to mix the following solutes. At first, 0.1575 mol (28.65 g) V_2_O_5_ (Sigma, USA) was added, and then 180 ml H_2_O_2_ (Maclin, Shanghai, China) was added. V_2_O_5_ was dissolved after about half an hour. Next, 10 ml H_3_PO_4_ (85 wt. %, Aladdin, USA) was diluted to 20 ml, and 5.56 ml of the diluted H_3_PO_4_ was added to the V_2_O_5_ solution. Stirring continued for 2 h at room temperature until the residual H_2_O_2_ was fully decomposed. The final solution was moved to a beaker (noted as solution A). Another solution (solution B) was prepared using 1000 ml deionized water, 0.81 mol (116.64 g) MoO_3_ (Aladdin, USA) and 13.2 ml diluted H_3_PO_4_ in a flask. The solution was heated to boiling with magnetic stirring. After solution B turned to yellow, 200 ml solution A was added repeatedly until all the solution A was injected. The mixture was evaporated naturally to 150 ml and finally 0.30 mol/L P_3_Mo_18_V_7_ was obtained.

When the LCFC was operated in batch mode aiming to study the cell reaction and optimize the operational conditions, the prepared anolyte (kept at room temperature of 23‒25 °C after 2 h reaction with fuel at 95 °C) and the catholyte were separately circulated between their storage vessels (conical bottles) and the cell using peristaltic pumps (BT100-2J, LongerPump, Baoding, China). Accompanied with the cell reaction, the reduced POM-I and Fe^2+^ was oxidized and reborn, and the POM-II was reduced. The LCFC was also operated with continuous redox reaction in anolyte so as to study the degradation degree and rate of carbohydrates. The conical bottle containing the mixture of the anolyte and a type of carbohydrate was put in a water bath at 85 °C so that the redox reaction in the anolyte and the cell reaction proceeded continuously (with excess catholyte). Thus, carbohydrates could be possibly oxidized stepwise to CO_2_ and water.

### Characterize the performance of the LCFC

The performance of the LCFC only using POMs as the catalyst was first measured, and then the effects of several Lewis acids as co-catalyst were compared.

Linear sweep voltammetry analysis was carried out to check the performance of the LCFC. An electrochemical working station (CHI 600E, Chenhua Instrument, China) was applied to examine the I-V curve by scanning from open-circuit voltage to 0.01 V at the rate of 0.10 mV/s, and calculate the output power density of the LCFC.

### Measurement of biomass degradation and products

During continuous operation of the LCFC, total organic carbon (TOC) of anolyte was analyzed at intervals using a TOC analyzer (TOC-L, Shimadzu, Japan). The variation of TOC was used to evaluate the degradation degree of carbohydrates. The degradation degree (DD) was calculated according to the following equation.

1 $${\rm{DD}}=({{\rm{TOC}}}_{0}-{{\rm{TOC}}}_{{\rm{t}}})/{{\rm{TOC}}}_{0}$$

where, TOC_0_ is the initial TOC of the anolyte, and TOC_t_ is the TOC at time *t*.

After a long-time operation, the final anolyte was collected and diluted by 500 times. The diluted solution was then filtered by 0.45 µm membrane (Jinteng, Tianjing, China) and the filtrate was analyzed using liquid chromatograph (1525HPLC, Waters, USA). The identified products were used to conjecture the possible degradation pathways of glucose.

### Measurement of electron transfer

To learn the mechanism of LCFC performance, the electron transfer from glucose to catalysts was first investigated using spectrophotometry and potentiometric titration, respectively.

For PMo_12_, the first step was to obtain the calibration curve. PMo_12_ solution of 1 mmol/L was reduced by 3 V electrochemical treatments for different times, so that a series of mixture solutions of Mo^5+^ and Mo^6+^ at different concentrations were obtained. The concentration of Mo^5+^ can be determined by titration using potassium permanganate, and it has a characteristic light absorption at 730 nm. So a calibration curve can be obtained and then used for measuring the conversion from Mo^6+^ to Mo^5+^. Thus, the electron transfer from carbohydrates to PMo_12_ can be calculated during thermal redox reaction.

The method of potentiometric titration was used to determine the electron transfer between PW_12_ and glucose. During thermal heating of the anolyte of PW_12_ and glucose, samples were taken out at interval times and the concentrations of W^5+^ were determined by titrated with potassium permanganate using an automatic potentiometric titrator (ZD-2, INESA, Shanghai, China).

### Data availability statement

All data generated or analyzed during this study are included in this published article.

## Results and Discussion

### Performance of the LCFC using POM as catalysts

The performance of the LCFC using POM as mono catalyst was first determined under several operational conditions including ambient temperature of cell reaction, initial concentrations of POM and carbohydrate, and flow rate of electrolyte solution (Table 1). These parameters were analyzed using four groups of batch experiments, and their general operational conditions followed the introduction in the section 2.1 except for some specified conditions.

**Table 1:** Operational conditions of the LCFC for the four groups of tests.

Parameters	a	b	c	d
POM-I type	PW_12_	PMo_12_	PMo_12_	PMo_12_
POM-I concentration (mol/L)	0.30	0.30	0.30	0.30
Temperature (°C)	variable	25	25	25
Phosphoric acid (mol/L)	3.0	variable	3.0	3.0
Flow rate of anolyte (ml/min)	27.3	27.3	variable	27.3
Initial concentration of glucose (mol/L)	1	1	2	variable

**Figure 2:**
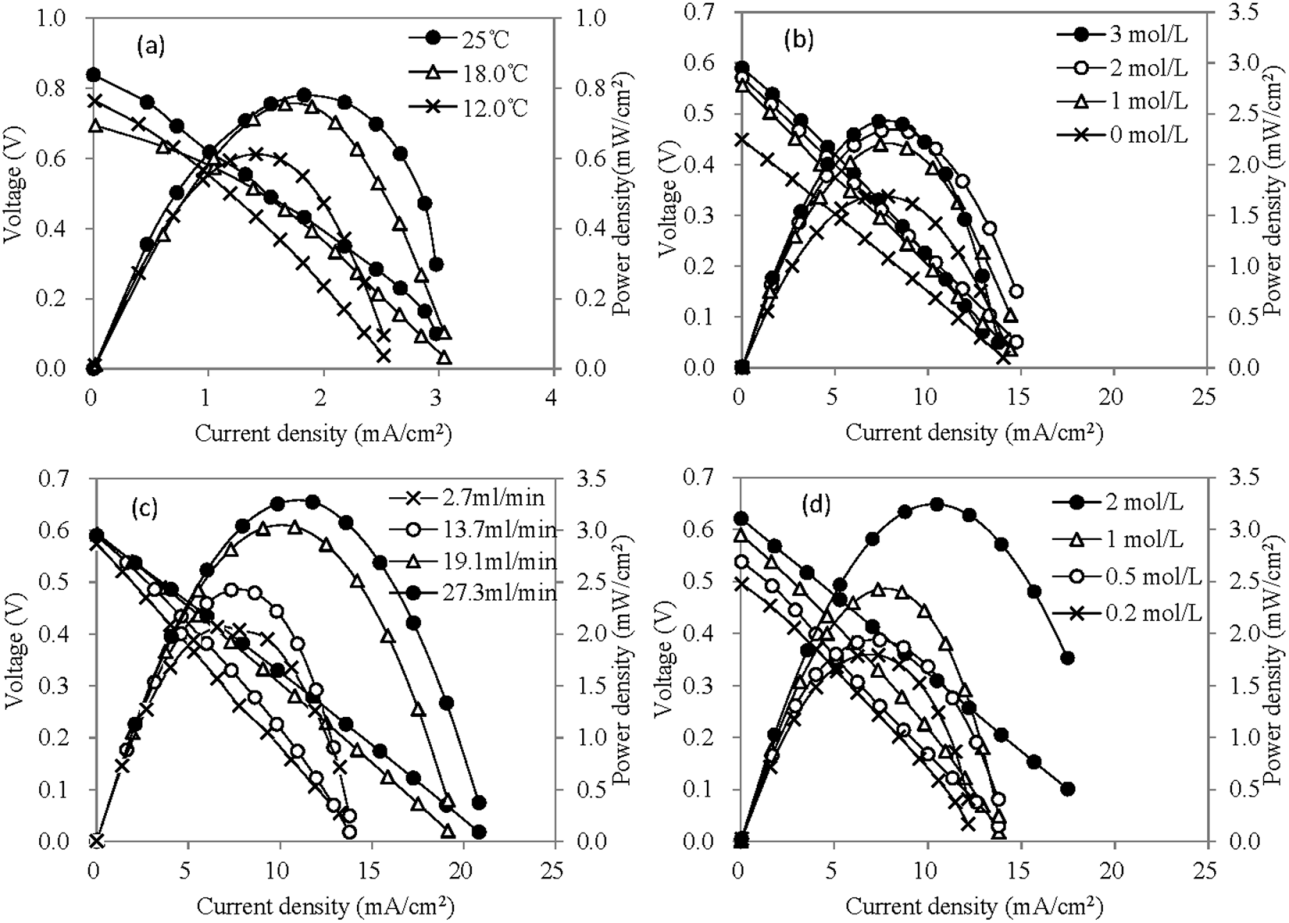
Performance of the LCFC under different operational conditions (**a**) using PW_12_ as POM-I at different temperatures; (**b**) using PMo_12_ as POM-I at different concentrations of phosphoric acid; (**c**) using PMo_12_ as POM-I at different flow rates of anolyte; (**d**) using PMo_12_ as POM-I at different initial glucose concentrations).

The fluctuation of ambient temperature impacted the output power density of the LCFC [Fig. 2a], because higher temperatures can accelerate the redox reaction between POM-I and POM-II in the cell. The maximum power density of LCFCs ever reported to be 40 mW/cm^2^ at high cell reaction temperature of 80 °C ^12^. On the other hand, the oxidation of biomass was carried out at a hot water bath, since this reaction was quite slow at ambient temperature and only slight color change of the anolyte was observed in 30 minutes. The similar phenomenon was also reported without heating condition^12^. The temperature for POM catalyzed reactions was usually higher than 60 °C ^19–22^, and thus the oxidation of biomass was carried out in a water bath of 85‒95 °C while the cell reaction run at ambient temperature of 23‒25 °C in the following tests.

The reduced POM-I released electrons in the carbon felt and at the same time protons passed through the proton exchange membrane from the anode chamber to the cathode chamber. Without phosphoric acid, the pH of the anolyte was approximately 0.5 and the cell reaction was normal. The addition of phosphoric acid increased the proton concentration in the anolyte and enhanced the proton transfer rate, and accordingly increased the out power density as shown in Fig. 2b. Moreover, PMo_12_ was very stable with phosphoric acid in solution^12^. However, when phosphoric acid concentration reached a high level of 2.0 mol/L, the further increase of phosphoric acid concentration cannot push the out power effectively. It was mainly attributed to high concentration of phosphoric acid would increase the viscosity of the solution and the corresponding internal resistance of the cell. Therefore, at most 3.0 mol/L phosphoric acid was applied to the LCFC.

Furthermore, the output power density was also improved with the increasing flow rate of anolyte [Fig. 2c], because more reduced POM-I flowed past the proton exchange membrane and the 3D extended electrode in a certain time. In fact, the maximum output power density was almost proportional to the flow rate of anolyte. The results also indicated that the release of electron from reduced POM-I was relatively faster than the oxidized POM-I obtaining electron from carbohydrates. Hence, it is important for the optimization of LCFCs to accelerate the reaction between electron shuttles and carbohydrate and enhance the contact of electron shuttles and electrodes.

The initial concentration of glucose in the anolyte would directly determine the output power density, as shown in Fig. 2d. The elevation of the initial glucose concentration improved the output power density significantly, and the relation between glucose concentration and the maximum of output power density almost followed an apparent linear type. During the same reaction time, the reduction rates of POM-I almost kept unchanged and thus higher glucose concentration produced more reduced POM-I for the subsequent cell reaction. However, excessive high concentration would result in the precipitation of glucose, and accordingly increase the internal resistance and even obstructed the cell.

Under these optimal conditions, the maximum power density reached 3.4 mW/cm^2^ at cell reaction temperature of 25°C. The following tests on the usage of Lewis acids would refer to these conditions. On the other hand, toward the further development of LCFCs in the field of biomass waste utilization, it is of great value to search highly-efficient catalysts, reduce the usage of phosphoric acid and toxic metals, lower the reaction temperature to ambient condition and improve the treatment efficiency. POMs can act as effective homogenous catalysts for hydrolysis of carbohydrates, while Lewis acids can also help the cleavage of glycosidic bonds^17, 23, 24^. Thus, a new type of LCFC was proposed, which used Lewis acids to replace most Bronsted acids.

### Improve LCFC by using ferric chloride to replace most POM-I

**Figure 3:**
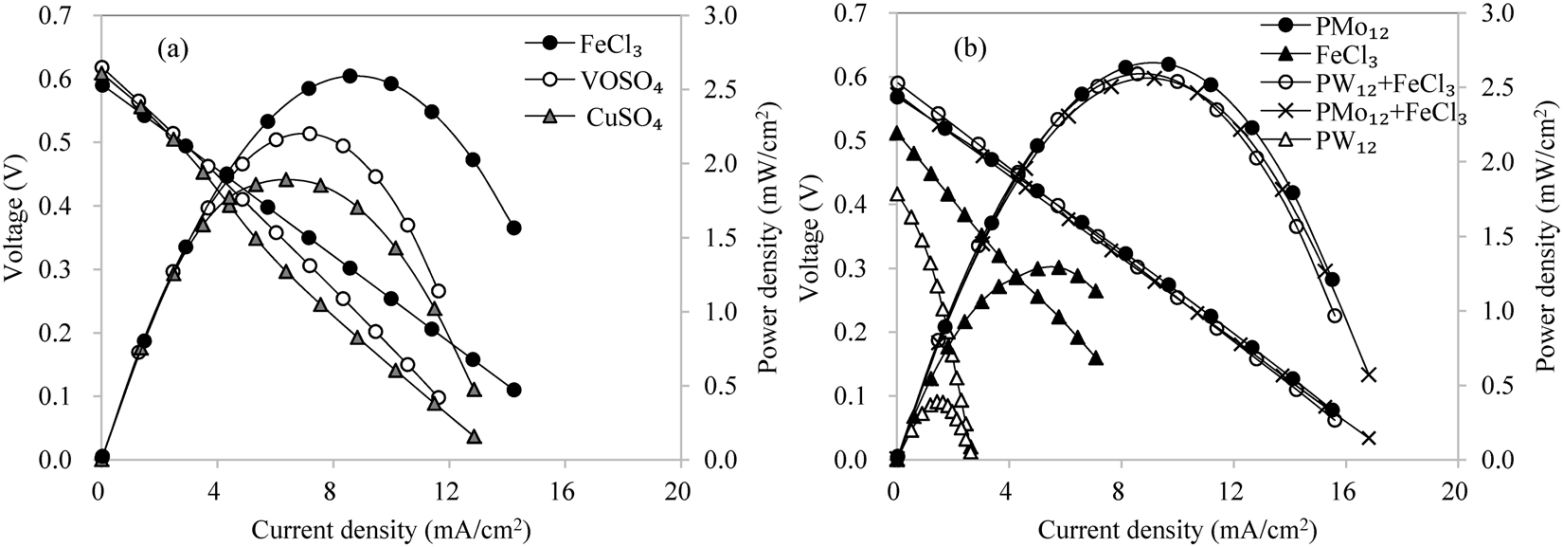
Performance of the LCFC using Lewis acid as co-catalyst (**a**) different Lewis acids; (**b**) combinations of FeCl_3_ and PW_12_ or PMo_12_; the concentrations of glucose, phosphoric acid, Lewis acid, mono PW_12_ and mono PMo_12_ were 1.0, 1.5, 1.00, 0.30 and 0.30 mol/L, respectively, while the concentrations of PW_12_ and PMo_12_ were reduced to 0.06 and 0.03 mol/L, respectively, when combining with FeCl_3_).

Three Lewis acids were tested including FeCl_3_, VOSO_4_ and CuSO_4_. Their concentrations were all 1.00 mol/L (higher concentration may result in precipitation in anolyte), while the concentration of PW_12_ was only 0.06 mol/L. One mol per liter glucose was first oxidized at 95 °C for 2 h in anolyte, and then the solution was pumped into the cell recurrently at ambient temperature of 23 °C. The performance was recorded as Fig. 3a. The ion pair of Fe^2+^ and Fe^3+^ exhibited the best output power density. Moreover, FeCl_3_ is the cheapest and easily available. Thus, FeCl_3_ was used for the further tests.

Five conditions were analyzed including FeCl_3_ combining PMo_12_, FeCl_3_ combining PW_12_, PMo_12_, PW_12_ and FeCl_3_. Figure 3b showed that the LCFC had similar power densities when it used PMo_12_ (0.30 mol/L), PMo_12_ (0.03 mol/L) plus FeCl_3_ (1.00 mol/L) or PW_12_ (0.06 mol/L) plus FeCl_3_ (1.00 mol/L) as the catalyst, while the LCFC’s performance decreased significantly when using FeCl_3_ (1.00 mol/L) or PW_12_ (0.30 mol/L). The other doses were also tested and the corresponding performance was slightly lower than or close to that in Fig. 3b. The results verified that the replacement of POM-I by FeCl_3_ kept the LCFC at the same level. The ability of PW_12_ was much lower than PMo_12_ in the LCFC, but the addition of FeCl_3_ increased the whole performance significantly. Due to the reduced usage of PMo_12_ or PW_12_, LCFCs can be operated less toxically and more cheaply.

**Figure 4:**
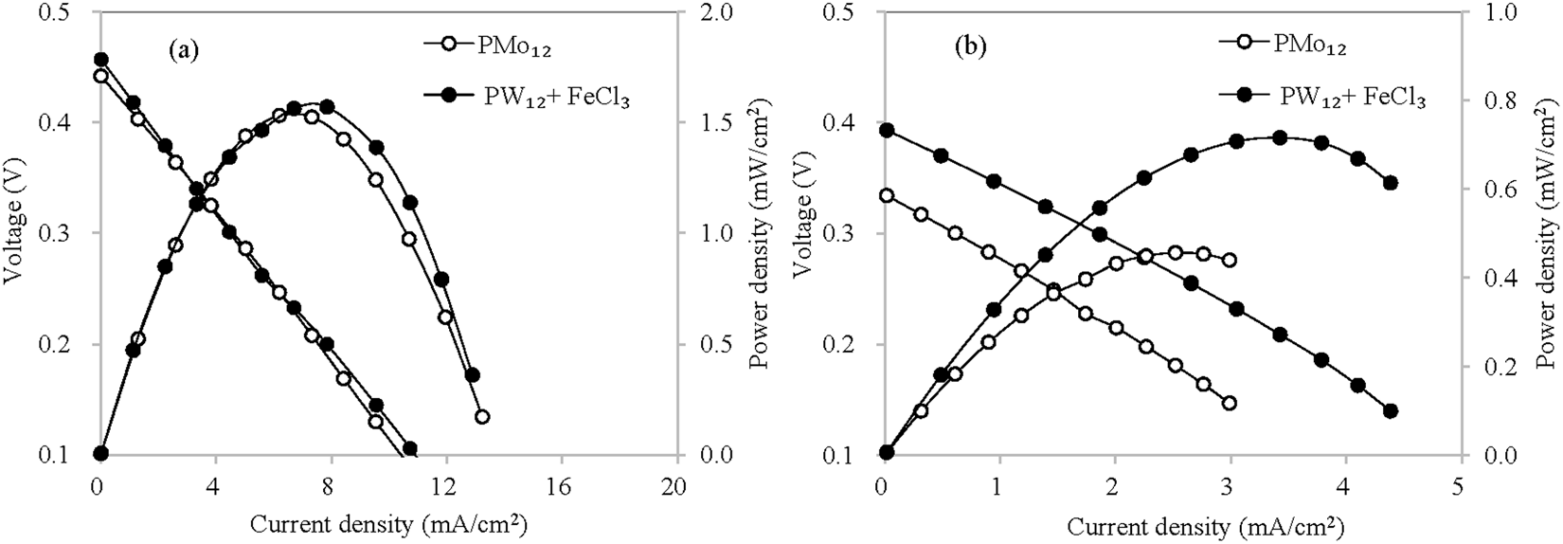
Performance of the LCFC using FeCl_3_ and PW_12_ as the catalyst when dealing with starch and cellulose (**a**) 40 g/L starch; (**b**) 40 g/L cellulose; PMo_12_, PW_12_, FeCl_3_ and phosphoric acid are 0.30, 0.06, 1.00 and 1.5 mol/L, respectively).

The improved LCFC can decompose glucose effectively. Furthermore, its capability was tested when dealing with polysaccharides including starch and cellulose (Fig. 4). When starch was used as the fuel, the thermal reaction in anolyte continued for 2 h at 95 °C; while cellulose was used as the fuel, the treatment duration was 6 h because of the weak degradability of cellulose. After that, the solution was pumped into the cell recurrently at ambient temperature of 23 °C. The results indicated that the improved LCFC can obtain the same performance with the raw LCFC only using PMo_12_ as catalyst when they utilized starch as the fuel. For cellulose, the added FeCl_3_ increased the maximum power density of the LCFC by 57% from 0.46 to 0.72 mW/cm^2^ and the open-circuit voltage by 18%. Starch is readily hydrolyzed compared with cellulose. The effect of Lewis acids on starch was not exhibited, while the added FeCl_3_ accelerated the hydrolysis of cellulose significantly. Besides that, the improved LCFC only need 20% of POM-I compared with the raw LCFC. Higher efficiency, safer chemicals and lower cost promise the new type of LCFC a great prospect in utilizing biomass waste.

For the LCFC only using FeCl_3_ as the catalyst, the reactions were concluded as following.

Anode: ${{\rm{C}}}_{{\rm{6}}}{{\rm{H}}}_{{\rm{12}}}{\rm{O}}{}_{{\rm{6}}}+{{\rm{6H}}}_{{\rm{2}}}{\rm{O}}+{{\rm{24Fe}}}^{3+}\to {{\rm{6CO}}}_{{\rm{2}}}\uparrow +{{\rm{24Fe}}}^{2+}+{{\rm{24H}}}^{+}$ (redox reaction when heating)

${{\rm{24Fe}}}^{{\rm{2}}+}-{\rm{24}}e\to {{{\rm{24Fe}}}_{{\rm{3}}}}^{+}$ (cell reaction at anode at ambient temperature)

Cathode: $${{\rm{6O}}}_{{\rm{2}}}+{\rm{24}}e+{{\rm{24H}}}^{+}\to {{\rm{12H}}}_{{\rm{2}}}{\rm{O}}$$

For practical application, the current open-circuit voltages (OCVs) were still low, possibly because the crossover of degradation products of biomass or ferric ions through Nafion membrane. To solve the problem, new types of proton exchange membrane could be applied in the future. For example, Sn_0.9_In_0.1_P_2_O_7_-based composite electrolyte membranes exhibited better performance than Nafion 114 in a metal redox fuel cell^25^.

### Complete degradation of carbohydrates

**Figure 5:**
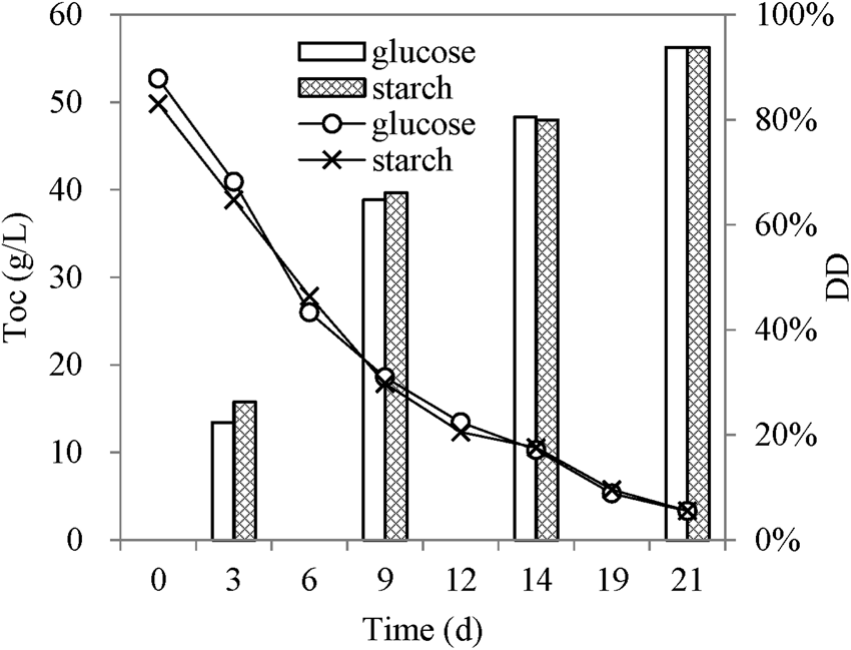
Variation of TOC in the anolyte (0.30 mol/L PMo_12_) when the continuously-operated LCFC consumed glucose or starch.

To learn the degradation process of carbohydrates in the LCFC, continuous experiments were carried out using both glucose and starch. The anolyte containing glucose or starch was kept in a water bath of 85 °C. As a consequence, the oxidation of biomass and the regeneration of reduced POM-I could take place simultaneously. The LCFC was operated continuously for a relative long time (21 days) and TOC in the anolyte were recorded. Along with the consumption of glucose, the concentration of raw PMo_12_ (yellow) in the anolyte decreased gradually and the color of the solution turned to blue (reduced PMo_12_). At the end, PMo_12_ was finally regenerated and the color of the anolyte recovered to yellow. The decrease of TOC in the anolyte verified a conversion from soluble biomass (glucose or starch) to carbon dioxide that escaped to air (Fig. 5). In 21 days, more than 93% of glucose and starch were completely decomposed in the LCFC. The degradation of glucose or starch means the release and transfer of electrons. The redox reaction between POM-I and glucose or starch was relatively slow compared with the subsequent cell reaction. From this point of view, the current density of the cell should be determined by the degradation rate of glucose or starch. Furthermore, the degradation rates of glucose and starch were almost the same, indicating the hydrolysis of starch to glucose was not the rate-limit step while the oxidation of glucose was relatively slow. Although the hydrolysis rate of cellulose was obviously slow (Fig. 4), the hydro lysate of cellulose is also glucose and it would be also oxidized completely in the continuous LCFC. Hence, the following part focused on the degradation of glucose.

**Figure 6:**
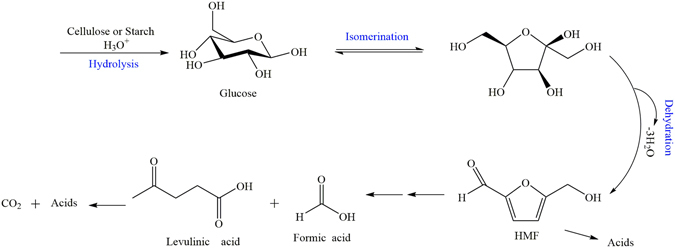
A possible reaction path of glucose oxidation in the LCFC.

Liquid chromatogram was used to analyze the final products. Referring to possible pathways (Fig. 6) and side reactions of glucose degradation^13, 17, 26^, nine key chemical compounds were detected including glucose, levulinate acid, formic acid, DL-glyceraldehyde, glycolic acid, dihydroxy acetone, pyruvate, D-erythrose and acetic acid. The results showed that there was no glucose in the final anolyte, indicating that all the glucose were hydrolyzed into small molecules or oxidized completely to CO_2_ and H_2_O. Among the eight possible intermediates and final products, only levulinate acid and dihydroxy acetone were detected and their concentrations were 4.5 and 25.8 mg/L, respectively. This suggested that acetic acid, formic acid and other compounds were fully consumed during the long time operation. The degradability of the residual compounds can be further investigated. Beside the pathway illustrated in Fig. 6, there existed some side reactions, which could be more complex. Nevertheless, this study verified that LCFCs can decompose macromolecular organic substances completely into CO_2_ and have great potential in the field of biomass waste treatment.

### Mechanism analysis on the effect of ferric chloride

Based on the results in 3.2 and 3.3, the degradation of starch or cellulose could be divided into two steps: hydrolysis to glucose and oxidation of glucose. The first step could be accelerated by FeCl_3_, especially for cellulose, as reported before^18^. For starch or glucose, FeCl_3_ plus a small quantity of PMo_12_ or PW_12_ also exhibited the same performance with single PMo_12_ (Figs 3 and 4). This illustrated that ferric ions played a similar role with PMo_12_ and PW_12_, including catalyzing hydrolysis, catalyzing glucose oxidation and electron shuttle.

**Figure 7:**
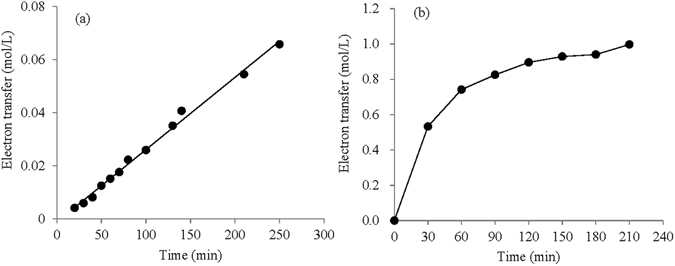
Numbers of electron transfer during redox reaction of PW_12_ (**a**) or PMo_12_ (**b**) and glucose (The concentrations of PW_12_ and glucose were 0.3 and 2 mol/L, respectively; the concentrations of PMo_12_ and glucose were 0.1 and 0.6 mol/L, respectively; the concentration of phosphoric acid in anolyte was 1.5 mol/L).

The output power density of the LCFC was decided by two factors. One was the quantity of electron transfer from biomass to catalyst during thermal redox reaction, and the other is the transfer efficiency of electrons and protons from anolyte to POM-II in the catholyte. The former can be enhanced by increasing the concentration of biomass and selecting suitable catalyst; the latter can be improved by adding acid and electron shuttles in anolyte or enlarging the effective area of electrodes. For the first step, the electron transfer from glucose to POM-I can reflect the oxidation degree of glucose, and also indicate the performance of catalysts oxidizing glucose. In fact, the efficiencies of PMo_12_, PW_12_ and FeCl_3_ varied greatly (Fig. 3b). The concentration of Mo^5+^ or W^5+^ in the anolyte was tested, and then the electron transfer from glucose to them was calculated (Fig. 7). The redox process between PW_12_ and glucose follows a zero-order reaction, indicating that both reactants were excessive during the reaction period and the rate of electron transfer was independent of the concentrations of PW_12_ and glucose. PMo_12_ showed a higher rate of electron transfer, which was nearly 40 times the rate of PW_12_ in the initial 1 h. According to the original concentration of Mo^6+^ and the electron transfer numbers, it can be deduced that nearly 83% of Mo^6+^ (1.20 mol/L) was reduced to Mo^5+^ (1.00 mol/L) in 210 min. On the other hand, it was obvious that more than one electron was given by one glucose molecule. The number of electron transfer during thermal redox reaction of POM-I and glucose would have a direct influence on the output power density in the subsequent cell reaction since more electrons transfer from glucose to POM-I resulted in higher concentration of reduced POM-I in the anolyte.

**Figure 8:**
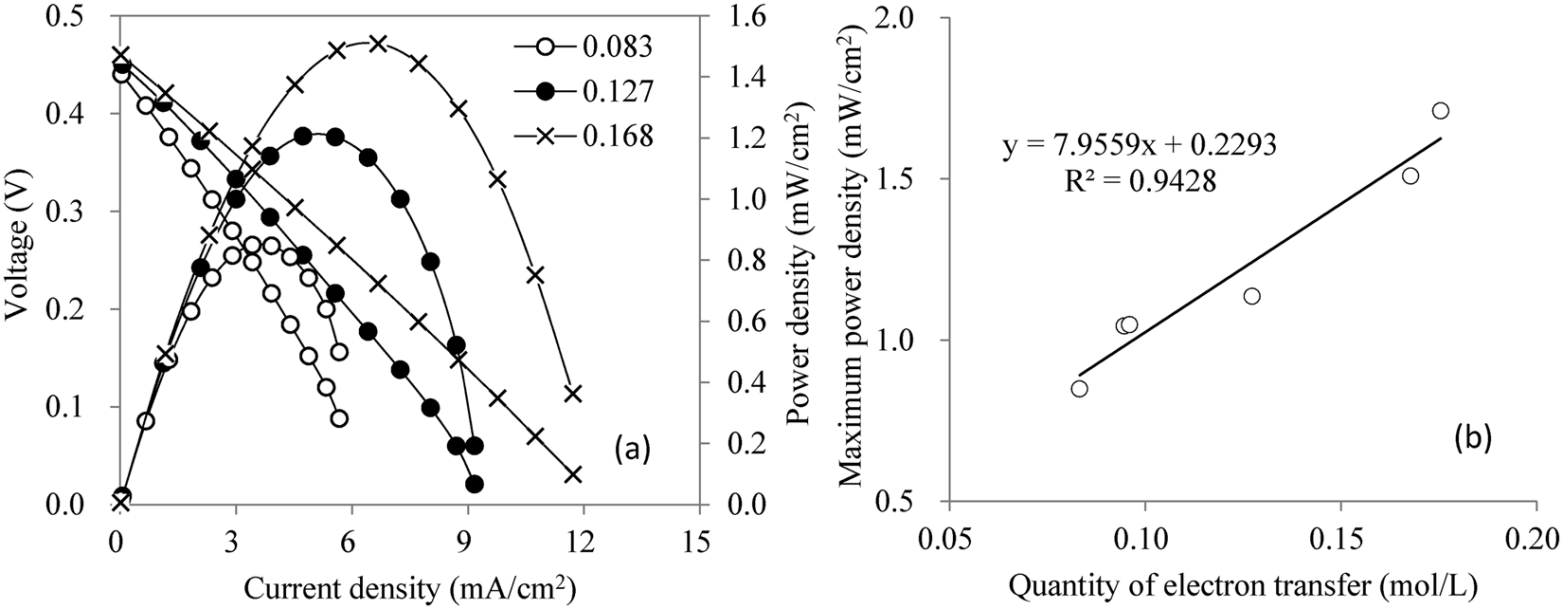
Performance of the LCFC using PW_12_ with different reduction degrees (**a**) performance corresponding to the electron transfer numbers of 0.083, 0.127 and 0.168 mol/L, respectively; (**b**) the relation between maximum power density and the numbers of electron transfer).

Further tests were carried out to verify this conjecture. The performance of the cell was tested when using PW_12_ with different reduction degrees as POM-I, which was obtained by setting different reaction times in the anolyte. The results showed that the output power density of the LCFC was directly related to electron transfer and the maximum power density had an approximate linear relation with the electron transfer number, as shown in Fig. 8. In addition to this proof, the maximum power density using PMo_12_ (0.30 mol/L) as POM-I reached 2.65 mW/cm^2^, which was about 6.8 times higher than that using PW_12_ (0.30 mol/L), as shown in Fig. 3b. Mo^6+^ has a relative high redox potential than W^6+^, and PMo_12_ had much faster rate of obtaining electrons from glucose than PW_12_, although molybdenum and tungsten are both belonging to one family.

**Figure 9:**
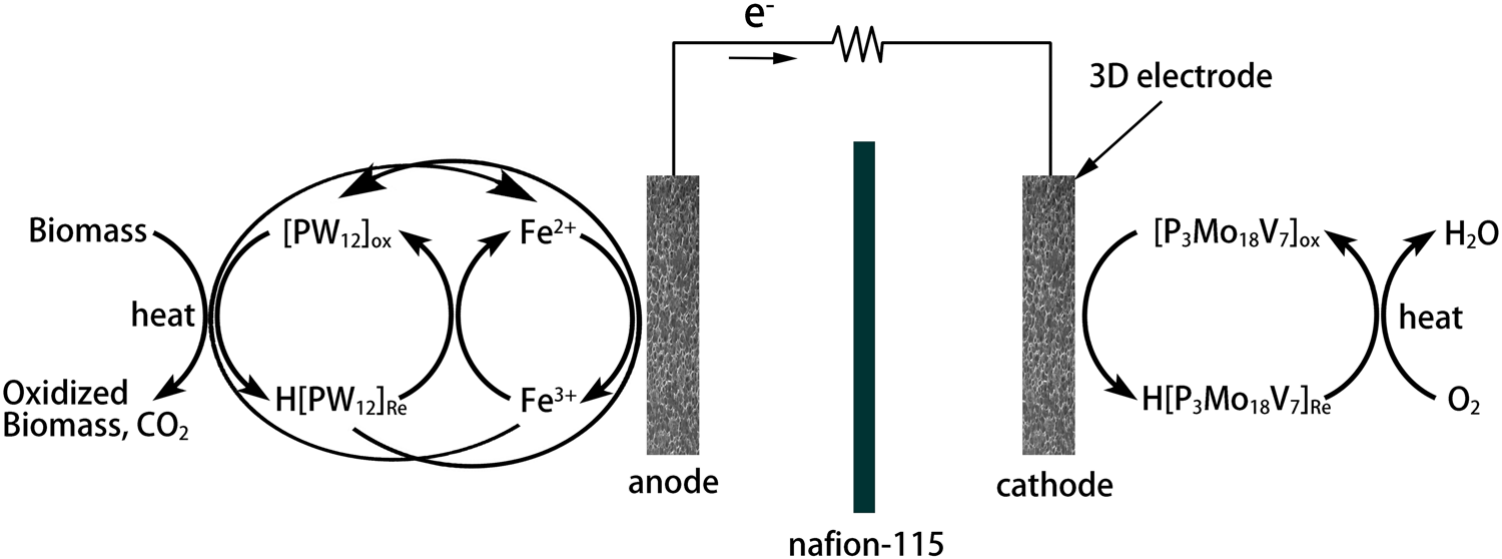
The schematic diagram of FeCl_3_ function in LCFC.

These results verified that obtaining electrons from carbohydrates and then transferring them to anode were the two main functions of catalysts in LCFCs. Considering the performance in Figs 3b and 4, it was deduced that the added FeCl_3_ not only contributed to the hydrolysis and oxidation of carbohydrates but also involved in the electron transfer in cell reaction. The redox couple of Fe^2+^/Fe^3+^ had a high solubility and proper electrochemical activity^27, 28^. Although PW_12_ had a relatively low rate of obtaining electrons, the added FeCl_3_ obtained electrons from PW_12_ and consequently stimulated the oxidation of carbohydrates by PW_12_. On the other hand, the cell with low concentration of PW_12_ (0.06 mol/L) and 1.00 mol/L FeCl_3_ reached the maximum power density of 2.59 mW/cm^2^ when using glucose as the fuel, which was very close to that using 0.30 mol/L PMo_12_. Obviously, Fe^2+^/Fe^3+^ also took part in the process of electron transfer directly. The functions of FeCl_3_ can be illustrated as Fig. 9.

## Conclusions

An improved liquid catalyzed fuel cell (LCFC) was developed for direct conversion from carbohydrates to electricity. Ferric chloride (FeCl_3_) was introduced into the LCFC as co-catalyst, which replaced 80% of the raw catalyst (polyoxometalates). The added FeCl_3_ accelerated the hydrolysis of carbohydrate and enhanced the electron transfer from carbohydrates to anode. As a consequence, the usage of polyoxometalates can be reduced by 80% and the performance of the LCFC kept at the same level. Moreover, the improved LCFC exhibited 57% better capability of utilizing cellulose as fuel.
